# Engineered nanoparticles for imaging and targeted drug delivery in hepatocellular carcinoma

**DOI:** 10.1186/s40164-025-00658-z

**Published:** 2025-04-30

**Authors:** Xianzhe Yu, Qin Zhang, Leibo Wang, Yan Zhang, Lingling Zhu

**Affiliations:** 1 Department of Medical Oncology, Cancer Center & Lung Cancer Center/Lung Cancer Institute, West China Hospital, Sichuan University, Chengdu, 610041 Sichuan People’s Republic of China; 2 Department of Gastrointestinal Surgery, Chengdu Second People’s Hospital, No. 10 Qinyun Nan Street, Chengdu, 610041 Sichuan People’s Republic of China; 3 Department of Postgraduate Students, West China School of Medicine/West China Hospital, Sichuan University, Chengdu, 610041 Sichuan People’s Republic of China; 4 Department of Surgery, Beijing Jishuitan Hospital Guizhou Hospital Guiyang, Guiyang, 550000 Guizhou The People’s Republic of China

**Keywords:** Hepatocellular carcinoma, Nanomedicine, Nanoparticles, Drug delivery, Tumor imaging, Early diagnosis

## Abstract

Liver cancer, notably hepatocellular carcinoma (HCC), poses a significant global health burden due to its high fatality rates. Conventional antitumor medications face challenges, including poor targeting, high toxicity, and drug resistance, leading to suboptimal clinical outcomes. This review focused on nanoparticle use in diagnosing and delivering medication for HCC, aiming to advance the development of nanomedicines for improved treatment outcomes. As an emerging frontier science and technology, nanotechnology has shown great potential, especially in precision medicine and personalized treatment. The success of nanosystems is attributable to their smaller size, biocompatibility, selective tumor accumulation, and lower toxicity. Nanoparticles, as a central part of nanotechnology innovation, have emerged in the field of medical diagnostics and therapeutics to overcome the various limitations of conventional chemotherapy, thus offering promising applications for improved selectivity, earlier and more precise diagnosis of cancers, personalized treatment, and overcoming drug resistance. Nanoparticles play a crucial role in drug delivery and imaging of HCC, with the body acting as a delivery system to target and deliver drugs or diagnostic reagents to specific organs or tissues, helping to accurately diagnose and target therapies while minimizing damage to healthy tissues. They protect drugs from early degradation and increase their biological half-life.

## Introduction

The global prevalence of liver cancer has been increasing, making it a significant health concern [1], with the annual incidence anticipated to exceed > 1 million individuals by 2025 [2]. Hepatocellular carcinoma (HCC) accounts for approximately 80–90% of primary liver cancer patients, making it among the most prevalent forms [2]. Due to challenges in early detection, most HCC patients are diagnosed at an advanced stage, characterized by high malignancy and rapid progression, posing significant treatment challenges [3]. Therefore, there is an urgent need for new integrated scientific and technological tools for the diagnosis and treatment of HCC in clinical practice.

Cancer theranostics is an emerging medical term used to denote the integration of treatment and diagnosis, bridging the gap between diagnosis and treatment [4]. The advantages of theranostics include improving the efficacy of anticancer drugs by monitoring their effectiveness against a patient's tumor throughout and after treatment and providing personalized and targeted immediate treatment [5]. The advanced nanosystems with enhanced bioavailability, biocompatibility, and drug loading capacity have been developed for targeted cancer therapy to reduce toxicity and improve the targeting properties [6]. Nanotheranostics systems are typically multifunctional nanosystems that can carry and deliver active cargoes to sites of interest and provide diagnostic capabilities, thereby enabling early detection and destruction of cancer cells in a more selectively [7]. In particular, theranostic nanoparticles (NPs) have the ability to accumulate in cancer tissues selectively and exert therapeutic effects, have a long circulation half-life, enhance tumor disposal, and are biodegradable and interact with specific conditions or activities in the tumor microenvironment, thus ensuring improved efficacy [8]. However, current evidence on tumor nanotheranostics needs to be further strengthened with research on the microbiological environment of tumors and studies on stimulus-responsive nanodrugs and drug co-delivery using nanocarriers [9].

**Fig. 1:**
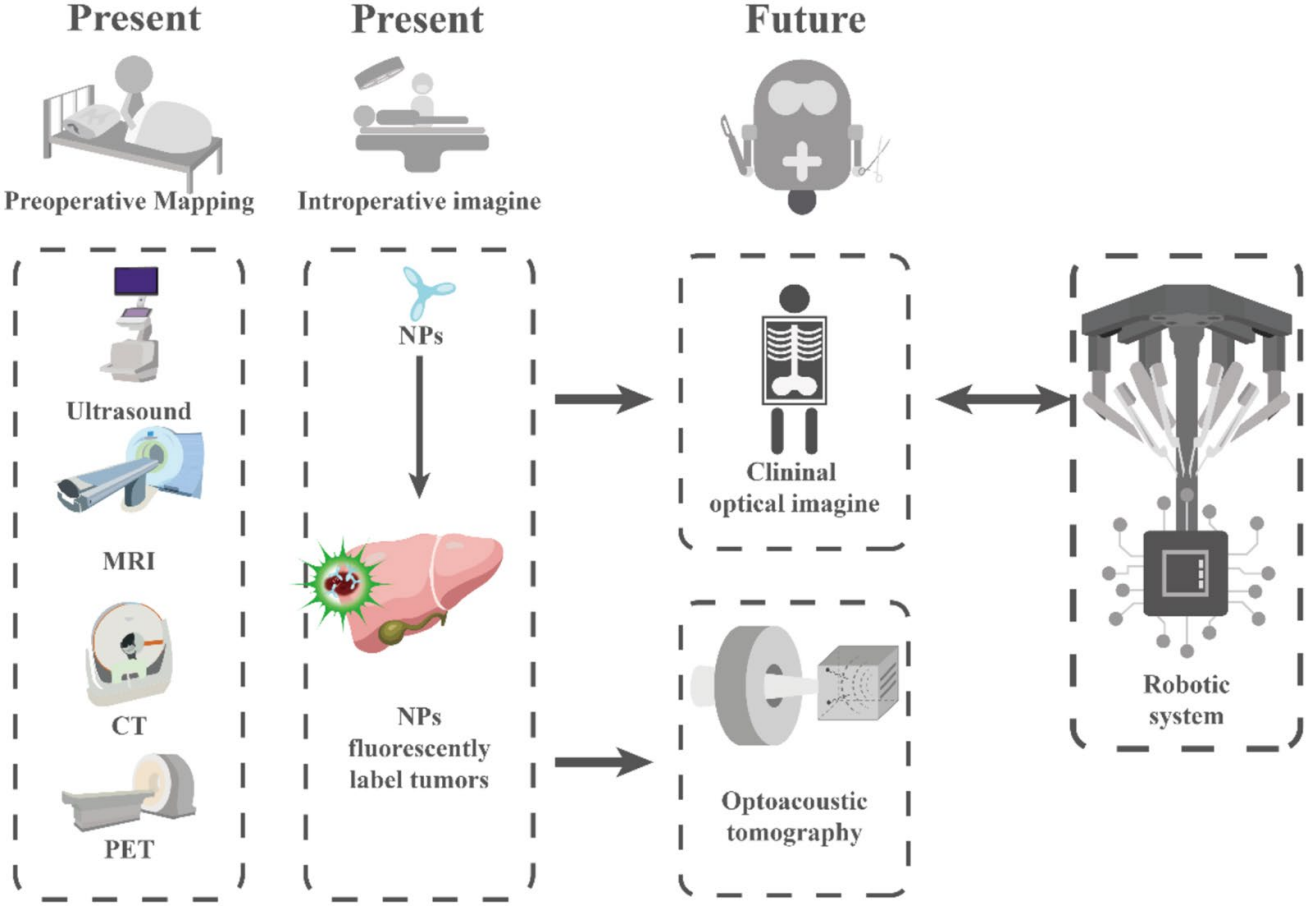
The current and future role of NPs in imaging-guided treatment in HCC. HCC: hepatocellular carcinoma; NPs: nanoparticles; CT: computed tomography; MRI: magnetic resonance imaging; PET: positron emission tomography; US: ultrasonography

A combination of magnetic resonance imaging (MRI), computed tomography (CT), and ultrasonography (US), which has traditionally been the primary method for diagnosing and screening HCC [10], often fails to detect cancer until substantial tissue changes have occurred, potentially allowing the cancer to metastasize. Additionally, conventional imaging methods struggle to differentiate between cancerous and benign tumors [11]. With its increased accuracy and sensitivity, NP-assisted imaging can aid in early cancer detection, real-time metastatic surveillance, and precise tumor identification [12]. Furthermore, the location and boundaries of healthy vs. affected cells and the edges of tumor tissue may be visualized using NPs [13]. Ongoing research is endeavoring to develop an increasing number of optically active and targeted imaging agents, including particle-based probes, for intra-operative clinical use. These probes have increased sensitivity, specificity, and depth of penetration. When used with state-of-the-art multichannel fluorography systems, they are expected to significantly improve real-time molecular detection and treatment of early-stage disease with clinical-grade accuracy (Fig. 1). These innovative NP-based imaging methods may enhance the precision of available HCC diagnostic instruments, provide earlier illness identification, and offer more individualized treatment plans, thereby improving survival rates.

**Fig. 2:**
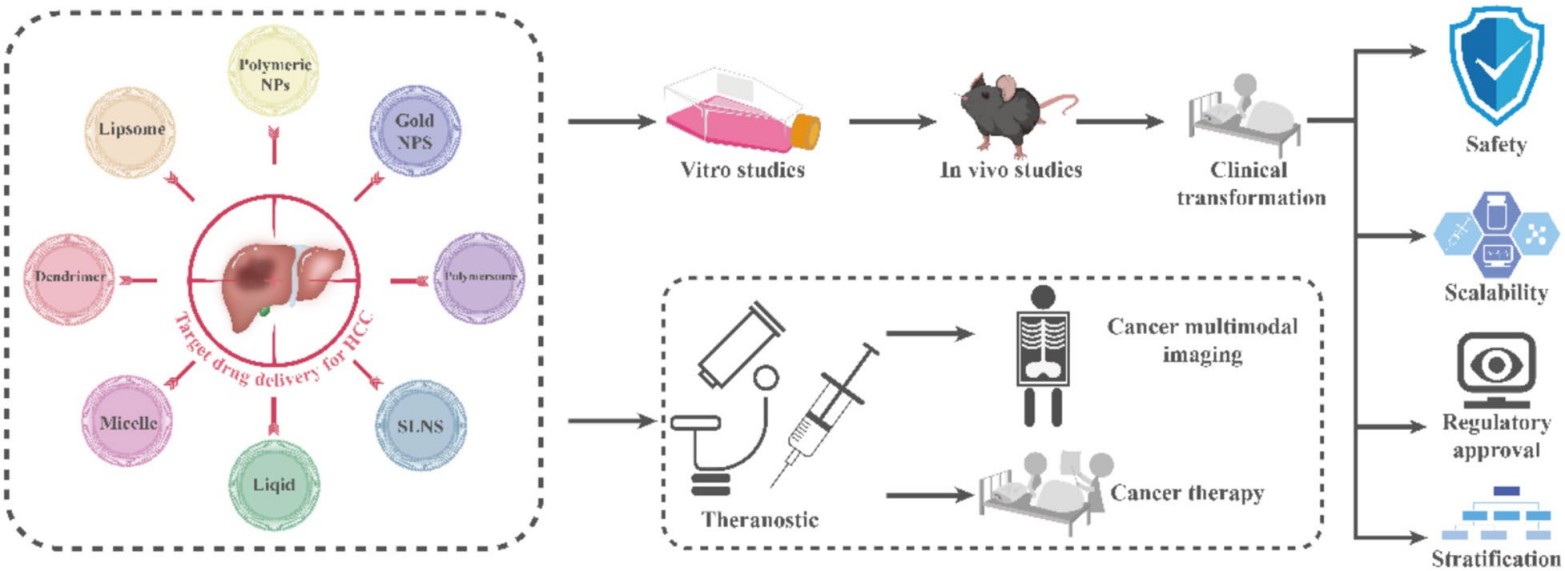
NPs for targeted drug delivery for hepatocellular carcinoma. HCC: hepatocellular carcinoma; NPs: nanoparticles; SLNS: solid lipid nanoparticles

Current therapeutic strategies for HCC aim to aggressively improve survival and treatment outcomes. They are typically used postoperatively or in combination with effective treatments [14]. NPs significantly improved bioavailability and enhanced therapeutic drug delivery, which are crucial for HCC treatment [15]. Many drugs are used to more effectively target liver cancer tissue by modifying the surfaces of drug-loaded NPs with peptides, small molecules, antibodies, vitamins, and other biological elements [16, 17]. By reducing the unintentional harmful effects of traditional therapies, NPs can significantly increase the effectiveness of conventional medications for eliminating tumors without endangering healthy cells [18]. Unlike traditional targeted drugs or chemotherapeutic carriers, recent advances in nanotechnology have focused on integrating their distinct properties with medications. These include liposome, polymer, biological, and metal NPs, which show promise for HCC identification and management [19] (Fig. 2). In recent years, much progress has been made in the application of NPs for the diagnosis and treatment of HCC [20]. This review aimed to assess the use of NPs for diagnosis and controlled drug delivery in HCC, with the intention to support the development of nanomedicines for HCC.

### Types and size of NPs

Based on their chemical composition, NPs are classified into three main categories: inorganic, organic and carbon-based [21]. Organic particles include polymer NPs, dendritic polymers, micelles, liposomes, lipid-based NP, and ferritin [22]. Liposomes are biocompatible, biodegradable, non-toxic, non-immunogenic carriers of active substances with hydrophilic and hydrophobic properties that are well known for their clinical applications [23]. The main advantages of liposomes are that liposomal drug delivery does not affect non-targeted healthy cells near the drug target because they are securely encapsulated in the liposomal core, and that the duration of the drug activity is increased by prolonging the drug's half-life and controlling the release of the drug [24]. Polymer-based liposomes improve drug effects, including reducing drug penetration into normal organs, protecting the activity of natural compounds, and increasing the cytotoxicity of drug compounds on tumor cells [25]. However, liposomes have low solubility and short half-life; it also requires a high production cost and is susceptible to oxidation of phospholipids.

Polymeric nanomaterials have been proposed as alternatives to liposomes owing to its good drug-carrying properties, controllable particle size, biodegradability, and prolonged drug circulation time in the body [26]. Polymeric NPs are biodegradable and have remarkable physical and mechanical properties; they become a topic of interest in a number of areas because of their ability to modify drug activity, delay and control drug release, increase drug adhesion, or enhance site-specific drug delivery [27].The disadvantage of polymer NPs is that drug release is usually biphasic and uncontrolled [15]. Organic NPs are generally more biocompatible than heavy metals, highlighting the importance of developing synthesis strategies that prioritize biocompatible and biodegradable NPs.

Inorganic NPs exhibit high chemical stability and corrosion resistant under physiological conditions. These include metal and metal oxide NPs, which possess unique magnetic, electrical, and optical properties that render them suitable for therapeutic diagnostic applications [28]. Inorganic NPs usually have high surface area-to-volume ratios and can be easily modified by surface conjugation chemistry, making them easy to prepare chemically [29]. These properties enhance their functionality in imaging, drug delivery, and therapeutic applications and provide additional advantages in ablating malignant tumor tissue [30, 31]. However, limitations such as high toxicity, non-biodegradability, accumulation in vital organs, high cost of large-scale production, and particle aggregation have prevented many inorganic NPs from being translated into clinical applications [32]. Compared with organic NPs, inorganic NPs exhibit more stable properties and can be reused multiple times. Moreover, these properties can be converted to each other, which enriches the use of inorganic NP [33]. Usually, organic and inorganic NPs have their own advantages and limitations. Therefore, it has been suggested to fuse these two types of NPs to overcome most of the drug delivery barriers [34].

Carbon-based nanomaterials (CNMs) have received more attention than other materials in biomedical fields, such as cancer imaging and therapy. Although CNMs are biocompatible and have low toxicity, their interactions with cells and biomolecules vary depending on their size, shape, surface charge, chemical composition, and solubility [35, 36]. CNM has multifunctional properties such as surface-to-volume ratio, thermal conductivity, rigid structural properties that can be post-chemically modified, and excellent biocompatibility with higher drug-carrying efficiencies, making it ideally suited for the design of composites with targeting capabilities, low toxicity, and high drug efficacy [37, 38].

In addition to their types, the size of NPs also determines their ability to translocate in tissues and organs, cross biological barriers and enter cells, as well as affects their pharmacokinetics, biodistribution, and tumor penetration. The “ideal” size for receptor-targeted NPs as carriers of diagnostic and therapeutic drugs is 10 to 60 nm [39]. These NPs serve as efficient carriers for many chemotherapeutic drugs, nucleic acids, imaging molecules, photothermal therapies, phytochemicals, and other biomolecules; additionally, they have the inherent targeting ability to deliver their cargo to the tumor site [40].

### NPs in imaging

Recently, there has been a growing interest in NPs as a diagnostic and therapeutic tool, with NPs increasingly used in imaging for tumor stratification, staging, and treatment response management [13]. Although CT, MRI, and US remain the main clinical diagnostic methods for HCC, typically capable of detecting HCC lesions with diameters of approximately 2–3 cm, detecting HCC foci that are < 2 cm in diameter remains challenging [41]. The limitations of these imaging modalities include their non-specific distribution, fast elimination, poor pharmacokinetics, and unfavorable consequences [42]. NPs have the potential to significantly alter the conventional imaging paradigm and advance the field of molecular imaging by serving as multifunctional platforms for radionuclide, optical, ultrasonographic, or MRI [43]. NPs and carriers are potential methods for tumor diagnostics due to their unique size, controlled delivery methods, enhanced signal density, quantification, amplification, surface properties and enhanced permeability and retention (EPR) effects [44]. Notably, due to their high uptake into the liver, NPs have been extensively used for visualizing HCC [45]. Nanotechnology-based imaging probes can recognize biomarkers on the surface of target cells, which are subsequently combined with the imaging performances of the NPs to facilitate accurate tumor localization in vivo [46].

In US imaging, NPs can be integrated into microbubbles and used as ultrasonographic contrast agents. These NP-encapsulated microbubbles are stabilized and echogenic, allowing them to release drug-containing NP payloads when triggered by ultrasound [47]. Inorganic NPs are often used in CT and MRI contrasts, which contain supermagnetic iron oxide NPs (SPIONs), which have been approved for MRI imaging of the liver [48]. Acetylated polyethyleneimine (PEI)-coated AuNPs were employed for negative CT imaging of HCC in the absence of antifouling modifications; CT contrast enhancement was much stronger in normal livers, whereas it was less effective in regions of hepatic necrosis induced by HCC [49]. Kim et al. [50] explored Mn_2_C-doped silica NPs as a liver-specific MRI contrast agent to improve HCC lesion visibility. Glucose-based modified dendritic polymer-coated gold NPs (Au DENPs), labeled with radionuclide 68 Ga and conjugated with cytosine-guanine oligonucleotides, were developed for dual-mode PET/CT imaging of tumors [51]. Porphyrin-based nanoparticles (68 Ga-F127-TAPP/TCPP(Mn) NPs) have been designed as PET/MRI dual-modal probes for lymph node metastasis imaging. Among these, NPs-based PET are considered the most effective for detecting distant metastases [52].

Disheartening HCC statistics show the urgent need for improving the early diagnosis of HCC to increase survival rates. Liu et al. [53] created a novel multifunctional polymeric NP contrast agent modified with both anti-vascular endothelial growth factor (VEGF) and gadolinium-diethylenetriaminepentaacetic acid antibodies for the early diagnosis of HCC. Gold nanorod@liposome core–shell NPs loaded with indocyanine green (ICG) were developed by Guan et al. [54] and exhibited excellent biocompatibility and high stability, proving the accuracy of the probe for early HCC diagnosis.

**Fig. 3:**
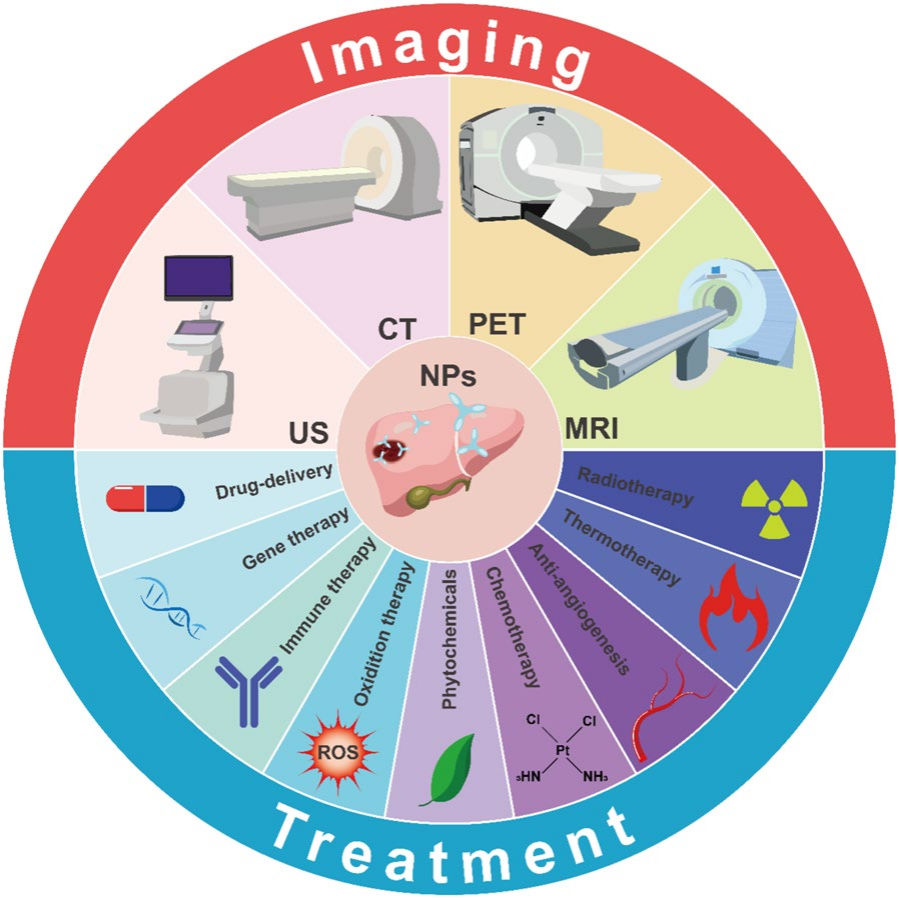
NPs in hepatocellular carcinoma imagine and treatment. CT: computed tomography; MRI: magnetic resonance imaging; NPs: nanoparticles; PET: positron emission tomography; US: ultrasonography

Intraoperative imaging methods aim to visualize the margins of HCC with greater accuracy and directly detect microscopic lesions not visible using other methods [55]. Wu et al. [56] combined organic electrochromic materials with near-infrared (NIR) photosensitizer NPs to develop a hepatic-cancer-targeting system for non-invasive real-time imaging of subcutaneous microscopic hepatic cancer in mice (diameter < 3 mm); the system could guide liver cancer surgery intraoperatively and precisely identify tumor margins in resected HCC tissues. Additionally, Ai et al. [57] developed long-lasting luminescent NPs that emit near-infrared light, guiding luminescence imaging in HCC surgery and facilitating pre-surgical CT imaging. This improved the excision of tumor tissues and the accuracy of HCC delineation in live mice. Research on NPs'role in HCC imaging is extensive (Fig. 3). These findings highlight significant advancements in integrating imaging with nanomedicine, enhancing our understanding of tumor-targeted drug delivery. This integration aids early detection and the development of personalized treatment plans for this aggressive disease.

### NPs for drug delivery in HCC treatment

Normal liver tissue receives 80% of its blood supply via the portal vein, and increased hepatic artery perfusion is a characteristic feature of HCC. Therefore, following systemic delivery, individuals with HCC experience reduced medication penetration into the liver due to reduced blood flow through the portal vein [58]. Meanwhile, sinusoidal fenestrations and Disse space act as biological barriers, restricting non-liver cells from entering the liver [59]. NPs offer a promising approach for successfully treating HCC as they navigate through the various biological and physical barriers that usually hinder traditional drugs. Interestingly, in the development of NPs for HCC, the liver serves not only as a target for NPs but also as one of the major barriers to NP delivery [60]. Additionally, NPs can protect drugs from premature degradation via encapsulation and the formation of complexes, which also have the advantage of enhancing drug selectivity and intracellular penetration into target tissues, as well as achieving controlled and sustained release to improve drug bioavailability [44, 61].

**Fig. 4:**
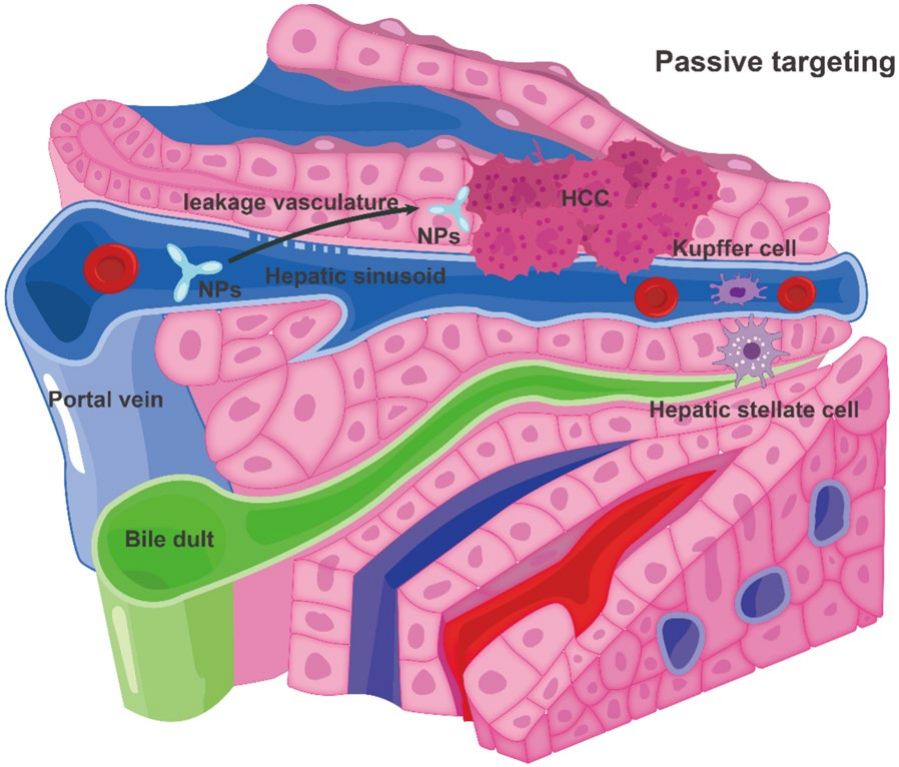
Passive targeting allows passage of NPs across leaky vasculature and subsequent accumulation in HCC tissues. HCC: hepatocellular carcinoma; NPs: nanoparticles

**Fig. 5:**
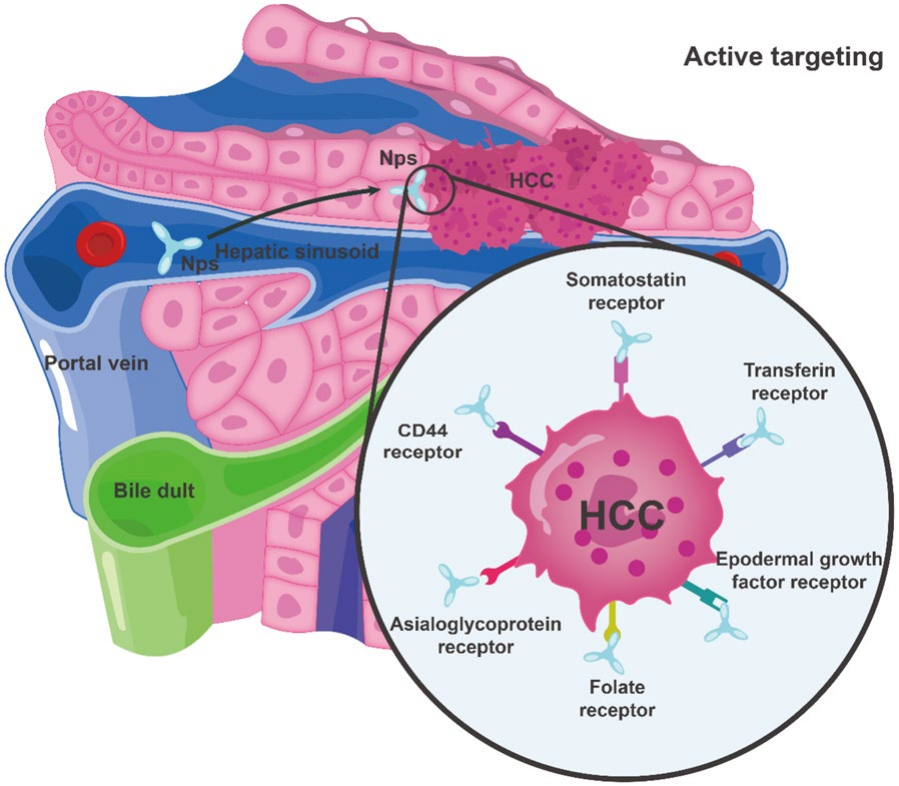
Active targeting strategies of NPs for liver. The modified NPs with specific ligands on the surface can be recognized by their receptors presents on a specific type of liver cells. NPs: nanoparticles

NPs can enter the liver through the hepatic portal triad. However, to avoid being trapped by Kupffer cells, NPs must first navigate through the biliary system [62]. Approximately, 60–90% of NPs are rapidly cleared from the circulation and accumulate in the liver, thereby increasing the concentration of drug in the liver and decreasing the amount of drug in the circulatory system, thus reducing adverse effects on other organs [63]. NPs are solubilized in the bloodstream, phagocytosed by macrophages, and accumulate in the organs of the reticuloendothelial system (RES); this passive targeting facilitates NP accumulation in the liver [64]. The passive targeting of NPs to HCC is associated with the EPR effect, which is mostly determined by their size, shape, surface charge, and biocompatibility [65]. The EPR effect can also be enhanced by wrapping NPs with cell membranes such as those derived from cancer cells, red blood cells, and platelets, enabling them to evade phagocytosis by macrophages [66]. In HCC, redox, enzyme, pH, heat, and light stimuli have been applied for the passive targeting of drug-carrying NPs [67]. Hepatic sinusoids have a robust blood supply and a large blood exchange region, which contribute to sluggish blood flow, a significant factor contributing to the ease of deposition of NPs in the liver. This phenomenon provides a good theoretical basis for the EPR effect of nanomaterials [68]. To achieve RES escape, the targeting ligands on the NP surfaces are altered to enhance the drug carriers'capacity to specifically target cells, reduce the hepatic barrier, and decrease drug toxicity, thus effectively releasing the drug to specific cells [69] (Fig. 4). Various surface receptors expressed on hepatocytes include desialylated glycoprotein, glycyrrhetinic acid, transferrin, folate, and integrin receptors [70]. Knowledge regarding the surface characteristics of NPs and functional groups present on the target molecule makes it possible to modify the surface of NPs by attaching targeting ligands, thereby obtaining target specificity and improving therapeutic efficacy [71]. Active targeting nanomedicines are internalized into cells through receptor-mediated endocytosis after surface modification through specific interactions, a common strategy for active enhancement of targeting in HCC [72] **(**Fig. 5**)**.

Many studies have demonstrated that NPs—which are classified as either inorganic, such as ceramic and metallic NPs, or organic, mostly structured macromolecules—can be effectively used to treat HCC [73]. Compared to chemotherapeutic drugs, the most prominent feature of NPs is their ability to deliver drugs specifically to cancer cells [74]. Chitosan NPs loaded with doxorubicin (DOX) and ginger extract exhibited remarkable efficacy against HCC in a diethylnitrosamine (DEN)-induced HCC model, significantly reduced tumor cell viability, and protected the heart from toxicity [75]. Ferulic-acid-conjugated chitosan NPs coated with glycyrrhizin were developed by El-Marakby et al. [76] for delivering ferulic acid to the liver and were proven as an effective and safe treatment for HCC. These NPs significantly increased cell death in HCC, indicating that nanotechnology could enhance the pharmacokinetic characteristics of current medicines. The architecture of mesoporous NPs allows for the local delivery of medications and particles to HCC that can accumulate and reach a gradient concentration in the tumor region. The aggregation of mesoporous NPs can effectively inhibit HCC [48]. These various advances suggest that NPs and co-loaded nanocarriers could be a new, safe and effective therapeutic and combination strategy for the treatment of HCC (Table 1).

**Table 1:** The Clinical trials for nanoparticles for hepatocellular carcinoma treatment

NCT	Phase	Study status	Type	Drugs that have been loaded	Number
NCT02314052	Phase 1/2	Terminated	Lipid Nanoparticle	DCR-MYC	21
NCT06309485	Phase 2	Not yet recruiting	Lipid Nanoparticle	WGI-0301	60
NCT04682847		Active, not recruiting	Iron Oxide Nanoparticle	Radiotherapy	40
NCT02716012	Phase 1	Active, not recruiting	liposomal Nanoparticle	MTL-CEBPA	75
NCT06689540	Phase 1	Recruiting	Lipid Nanoparticle	MTS105	14
NCT05497453	Phase 1/2	Recruiting	Lipid Nanoparticle	OTX-2002	190
NCT05451043	Phase 2	Recruiting	Nanoparticle albumin-bound	Durvalumab	62
NCT05092373	Phase 1	Recruiting	Albumin-Stabilized Nanoparticle Paclitaxel	Pembrolizumab	43

### Gene therapy with NPs

In HCC, gene therapies that regulate gene expression can be helpful, as the chemicals that impact RNA and DNA can efficiently change cell behavior [77]. MicroRNAs (miRNAs), together with small interfering RNAs (siRNAs), have been studied as nucleic acid-based medicines. miRNAs are non-coding, single-stranded molecules that act at the post-translational level to modify or silence RNAs [78], whereas siRNAs are double-stranded RNAs that can suppress gene expression via targeted degradation of antisense-stranded mRNA at several locations [79]. Due to enzyme-mediated gene degradation and their brief circulation time in the bloodstream, NPs find another application in cancer therapy: gene delivery. This approach safeguards nucleic acids from enzymatic degradation and facilitates intracellular chemotaxis [80]. The high polycationic properties, biocompatibility, low toxicity and efficient permeation properties make this nanoparticle and its derivatives a novel gene delivery system for HCC [81].

NPs loaded with siRNAs play a significant role in HCC treatment [82]. Rational optimization of NPs via modifications in their shape, size, and surface chemistry can increase the in vivo stability and encapsulation rate of siRNAs. Such optimized NPs can also inhibit the kidneys and the monocyte–macrophage system from clearing the siRNAs and protect siRNAs from nuclease degradation [83, 84]. Given their surface-modified active targeting capability, NPs silence genes efficiently at low dosages and prevent harmful effects by decreasing the accumulation of siRNAs in non-target tissues [85]. Using galactosylated chitosan PEG NPs, siRNA was delivered to the oncogenic gene polo-like kinase 1 in a HepG2 cell mouse xenograft model. The pro-apoptotic molecules p21, Bax, and p53 were upregulated, whereas the anti-apoptotic protein Bcl2 was downregulated in conjunction with substantial tumor regression [86]. A construct of cationic straight-chain starch NPs incorporating folic acid functionalized and loaded with SPIONs specifically targeted survivin, via siRNA, which effectively silenced survivin while inducing apoptosis in HCC cells in vitro [87, 88]. The half-life of siRNAs is increased by the modification of the attached SPIONs, which also shields them from nuclease destruction in biological systems and facilitates their entry into liver cancer cells without causing cytotoxicity in vitro [89]. Moreover, AuNPs modified with branched PEI were developed as effective and secure delivery systems for siRNAs into cells [90]. Lipid NPs carrying siRNAs adsorb apolipoprotein E on their surfaces and bind to the low-density lipoprotein receptor, facilitating absorption by HCC and hepatocytes [91]. Similarly, Wang et al. [92] developed a novel biodegradable cationic polymer based on disulfide linkages loaded with small interfering VEGF RNA with anti-angiogenic effects in HCC.

miRNAs attach to untranslated regions in mRNAs to control various functions, including angiogenesis, metastasis, and proliferation, making them an important therapeutic target in several cancers [93]. miRNAs are frequently dysregulated in HCC, and specific miRNAs are associated with HCC clinicopathologic features, including metastasis, recurrence, and prognosis [94]. NPs shield free miRNAs from negative charges that would otherwise prevent cellular uptake and reveal targeting ligands that are only triggered by particular environmental stimuli and can release the encapsulated miRNAs [95]. AuNP-miRNA-375 has a high cellular uptake rate, retains miRNA-375 activity, and may be utilized to treat HCC that is incurable; it has the ability to inhibit cell growth, invasion, migration, and colony formation, as well as induce death in HCC cells [96]. Adriamycin carbonate NPs encapsulated in lipids have strong cytotoxicity, enhanced accumulation, effectively transport miRNA-375, and foster synergistic effects in chemotherapy-resistant HCC cells [97]. Silicon oxide NPs to deliver Adriamycin and miRNA-375 to the HepG2 human hepatoblastoma cell line have been employed to overcome multidrug resistance (MDR) [96]. In another study, the encapsulation of polylactic-co-glycolic acid (PLGA) NPs with PEI and vector miRNA loading suppressed HepG2 cell proliferation [98]. The ability of peptide-based NPs to selectively distribute miR-199a-3p underscores their promise as a potential treatment approach for HCC [99].

Suicide gene therapy is a novel method to treat HCC that stands out in nanosystem delivery [100]. Wang et al. [101] developed shape-controlled magnetic mesoporous silica NPs, and their performance in MRI-guided, magnetically targeted and hyperthermia-enhanced suicide gene therapy of HCC was investigated. Superior therapeutic efficacy for HCC has been established through the encapsulation of PLGA-PEG-PEI NPs, which selectively strongly expresses suicide genes in tumors and leads to a decrease in tumor growth [102]. Moreover, polyamidoamine (PAMAM) dendritic macromolecules are widely used for gene delivery [103]. Apoptin was loaded onto ornithine-conjugated PAMAM and showed better transfection efficiency and intracellular uptake, which resulted in upregulation of apoptosis in HepG2 cells [104]. Inhibition of HepG2 xenografts in nude mice by DOX delivered via N-acetylgalactosamine-conjugated PAMAM provided protection against cardiotoxicity [105]. Future advances will allow the development of new formulations and NP-based solutions that can overcome the pharmacokinetic limitations of currently available drugs and improve therapeutic efficiency and outcomes.

### Immunotherapy with NPs

Cancer immunotherapy for HCC has emerged rapidly over the past decade because the liver is the main immunological organ of the lymphatic system and plays a vital role in HCC treatment [106]. Various targeting strategies, such as NPs that can target the cancer cells, tumor microenvironment (TME), or peripheral immune system components directly, are the foundation of NP-dependent cancer immunotherapies. These strategies allow NPs to overcome TME suppression and specifically target tumor cells while effectively decreasing damage to normal cells [107, 108]. Moreover, the surface of NPs is highly modifiable, enabling effective immune cell modulation. Binding moieties such as ligands, peptides, and antibodies to NP surfaces enhances targeting and significantly extends circulation half-life [109]. As most liver cancer cells overexpress folate receptors on their surface, Duan et al. [110] developed folate-modified chitosan NPs encapsulating the human interferon-γ-inducible protein-10 (*IP-10*) gene, which inhibited the growth of the tumor, extended survival in nude mice receiving subcutaneous transplantation of human HCC, and specifically bound to the folate receptor on HCC cells to facilitate the *IP-10* expression. This, in turn, increased the activity of particular cytotoxic T lymphocytes and efficiently enhanced interferon-gamma secretion.

NPs enhance the local efficacy of immune checkpoint inhibitors while minimizing off-target effects. Through in situ circulation, SPIONs linked to anti-PD1 antibodies are injected directly into HCC tumor. In HCC treatment, NPs modulate the immunosuppressive TME, synergistically suppressing tumor growth and activating immune responses [111]. Xiao et al. [112] coupled anti-PD-1 treatment with CXCR4-targeted p53 mRNA NPs, which successfully promoted complete reprogramming of the immunological TME's molecular and cellular components, thereby reversing immunosuppression in HCC. Notably, cell surface molecular targeting of NPs significantly enhanced the activation of CD8^+^ cytotoxic T lymphocytes [113]. These findings suggest that NPs can enhance immunotherapy for HCC.

Furthermore, NPs have been employed as vaccine carriers to strengthen the immune system and shield antigens from being destroyed by immune cells [114]. Combined HCC treatment using folate-chitosan/mouse IP-10 and DC/tumor cell fusion vaccination successfully extended survival and suppressed the development of implanted HCC tumors in mice [115]. To improve the anti-cancer immune response, Wang et al. [116] created a unique antigenic nanovaccine using photosensitive/acidic DCs that transformed tumor-related neutrophils. This nanovaccine facilitated the release of tumor-related antigens, killed immune cells, and specifically targeted H22 tumors, serving as an in situ tumor vaccine to boost anti-tumor T cell responses against primary H22 tumor growth. These various studies suggest that NP-based strategies can improve the efficiency and effectiveness of immunotherapy for HCC.

### Oxidation therapy with NPs

Compare with other cells, HCC cells have a higher oxidative status because of the higher concentrations of reactive oxygen species (ROS), which are cancer markers and potential targets for HCC therapy [117]. NPs can induce ROS production in HepG2 cells and activating the caspase-3 cascade, leading to cancer cell apoptosis [118, 119]. CeO_2_ NPs are cytoprotective to normal cells and cytotoxic to cancer cells; they can induce the production of ROS in cancer cells, which subsequently generates reactive nitrogen that disrupts intracellular activity, a characteristic that makes them an outstanding anticancer agent [120]. Jiang et al. [121] developed a pH-responsive nanoplatform (PEG-MSN@ATO) that elevated anti-tumor effectiveness in vivo by causing tumor cells to undergo apoptosis and produce ROS, preventing tumor cell growth and metastasis and triggering anti-tumor immunity inside the TME. Usmani et al. [122] prepared drug-carrying NPs that significantly reduced the survival of HepG2 cells and exhibited a high rate of apoptosis while being non-toxic and harmless to hepatocytes. These drug-carrier NPs stimulated intracellular ROS production, which may have been one of the reasons for the induction of apoptosis.

### Phytochemicals with NPs

Owing to their different biological activities (antioxidant, anti-inflammatory, anticancer, and immunomodulatory), phytochemicals are valuable sources of anticancer therapeutic molecules that have been widely investigated for their anticancer and hepatoprotective effects [123]. NP-based carrier systems enhance the delivery of these plant bioactives by controlling drug release, protecting unstable compounds, and improving bioavailability, solubility, and absorption [124].

Resveratrol (RSV) is a phytonutrient synthesized by and extracted from plants that has chemical sensitizing, antioxidant, and anti-inflammatory properties [125]. Moreover, RSV activates caspase-3 to cause apoptosis in cancerous liver cells, upregulating the Bax/Bcl2 ratio and increasing P53 expression [126]. Encapsulating RSV in NPs for cancer therapy has received substantial attention because it can improve drug accumulation in tumors by enhancing permeability and retention [127]. RSV, when encapsulated in glycyrrhizic-acid-coupled human serum albumin NPs, shows effective targeted delivery to and slow-release properties in HCC cells [128]. Rahman et al. [129] constructed cationic solid lipid NPs loaded with RSV that showed relatively high cytotoxicity and better anti-tumor activity against HepG2 cell lines.

Hesperidin (HP), a natural flavonoid in citrus fruits, has drawn much interest for its potential to prevent and promote cancer [130]. AuNPs conjugated with HP possessed better anti-inflammatory, anticancer, and anti-proliferative performance in DEN-induced HCC and had the capacity to modulate signaling pathways [131].

Chitosan, a deacetylated derivative of chitin, is a popular NP used to treat various illnesses. As a non-toxic, biodegradable, bioavailable, and biocompatible polymer, chitosan is widely used in medicine due to its immune-boosting, anti-fungal, anti-microbial, and anti-tumor properties [132, 133]. Furthermore, chitosan has a strong internalizing ability, increased cytotoxic activity, and major anti-inflammatory effects in HCC treatment by blocking the synthesis of cytokines, which are essential for hepatocyte survival [134]. Chitosan NPs containing ginger extract and DOX proved to be potent anti-HCC agents in vivo and in vitro, significantly decreasing tumor cell survival and protecting them from cardiotoxicity [75].

Turmeric, an Indian spice, contains a polyphenolic yellow component called curcumin (Cur). The rapid metabolism, low stability, and limited water solubility of Cur restrict its bioavailability [135]; however, it promotes apoptosis in HepG2 cells and suppresses the growth of several HCC cell lines by enhancing the generation of ROS and modulating the tumor growth factor-β1/Smad3 signaling pathway [136]. A recent study by Wu et al. demonstrated the efficacy of platelet membrane-coated CUR-loaded PLGA NPs in BALB/c fasted mice with HepG2 tumors. These NPs showed enhanced tumor-targeting ability and the highest potential for antitumor activity [137]. In another study, treatment of HCC mice bearing H22 cells with modified carrying curcumin and berberine (CURGL/BBR-HL) resulted in a lower tumor volume than that of both free drug and non-modified liposomal formulations, and a tumor growth inhibition of 68.56%, which was 68.56% higher than that of carrying curcumin and berberine (CURGL/BBR-HL) and carrying curcumin and berberine (CURGL/BBR-HL) by 1.34- and 1.85-fold, respectively [138].

Paclitaxel is a commonly used antitumor phytochemical drug. Owing to their rubber-like quality, which increases their receptor-mediated endocytosis and EPR by hepatocyte targeting ligands, RNA NPs carrying paclitaxel and miRNA122 were effectively transported to liver cancer cells [139]. Camptothecin is a plant-derived alkaloid that is widely used as an antitumor compound, including for HCC; however, its clinical use is limited by poor water solubility, low bioavailability, and severe toxic side effects [140]. Fu et al. [141] prepared galactose-modified trimethyl chitosan-camptothecin prodrug NPs, which inhibited premature camptothecin release and showed great potential in targeted chemotherapy for HCC.

Overall, these studies suggest that combining botanicals with nanotechnology enhances the effectiveness of anticancer medications by addressing issues such as low bioavailability and toxicity, making it a promising approach.

### Chemotherapy with NPs

Traditional chemotherapy for HCC has disadvantages such as MDR, severe side effects, high clearance rates, drug delivery to inappropriate HCC locations, and low concentrations that ultimately reach HCC cells, which greatly impede the clinical application of chemotherapeutic drugs [142, 143]. Various NP-encapsulated chemotherapeutic agents can improve therapeutic efficacy and mitigate the unfavorable effects of systemically administered chemotherapy by enhancing the biodistribution, pharmacokinetics, and accumulation of cytotoxic agents at the tumor site [144]. In addition to encapsulating chemotherapeutic drugs in nanomaterials for precise drug release at the HCC site, utilizing NIR rays, microwaves, and magnetic fields as external inducers to precisely release NPs is another possibility [145]. Lai et al. [146] reported the use of 5-fluorouracil-zein NPs for liver targeting, demonstrating its excellent stability, high drug-loading capacity, and slow release rate in vitro. These NPs exhibited the highest relative absorption in liver tissue, suggesting their favorable properties for hepatic-targeted drug delivery.

The chitosan-coated DOX NP delivery system inhibited HCC cell growth by promoting apoptosis via the p53/PRC1 pathway and arresting the cell cycle in the G2/M phase [147]. When utilized in HCC xenograft models, the comparatively high cellular absorption of DOX/lithocholic acid-poly(ethylene glycol)-lactobionic acid NPs in human HCC cell lines evidently suppressed the migration, invasion, proliferation, and angiogenesis of liver tumors in vivo [148]. Compared with DOX alone, dual loading of chitosan NPs generated marked anticancer effects on HepG2 cells by significantly reducing VEGF and B-cell lymphoma-2 secretion levels, stimulating antioxidant enzymes, and suppressing MDR in liver tissues [149]. The PLGA NPs coated with transferrin that contained the chemotherapeutic drugs cisplatin and DOX overcome MDR, improve safety, and consequently promote therapeutic efficacy [150].

Combined transarterial chemoembolization (TACE) and transarterial radioembolization therapy based on multifunctional NPs is expected to overcome drug resistance in hypoxic tumors and improve therapeutic efficacy [151]. In TACE for HCC, nanocarriers are essential for improving therapeutic efficacy and minimizing side effects [152]. Liu et al. developed a bimodal imaging nanoplatform (Fe_3_O_4_@PMO-Cy5.5) for TACE of HCC by incorporating periodic mesoporous organosilica (PMO) with Cy5.5 and Fe_3_O_4_ NP molecules. Fe_3_O_4_@PMO-Cy5.5 loaded with DOX may infiltrate HCC cells and inhibit their proliferation [153]. A hybridized composite of SPIONs encapsulating DOX has been created as a potentially effective therapeutic agent for HCC therapy by applying the TACE method [154]. The nanoplatform's distribution of chemotherapeutic drugs in local tumor tissues is enhanced, and it avoids the substantial side effects associated with systemic administration, which is the main benefit of TACE for HCC [155]. Transarterial administration of integrin inhibitor NPs combined with TACE significantly delays tumor growth and intrahepatic metastasis in an animal model of HCC [156].

### Anti-angiogenic therapy with NPs

HCC tumors have a large blood supply; thus, angiogenesis is crucial for their development. Anti-angiogenic therapies, which include the inhibition of kinases that promote tumor growth or increase its blood supply, are promising in HCC treatment [157]. Owing to the size gap in the tumor vasculature, similarly-sized nanostructured materials can be used to access the leaking tumor vasculature to accurately deliver loaded anti-cancer NPs to the tumor tissue [158].

Tang et al. [159] prepared d-α-tocopherol PEG 1000 succinate polycaprolactone (TPGS-b-PCL) NPs. HepG2 cell proliferation was more successfully suppressed with TPGS-b-PCL NPs loaded with sorafenib than with free sorafenib. Delivery of VEGF siRNA via NPs downregulated VEGF expression in HCC in vivo and in vitro and generated strong anti-angiogenic effects in the TME of a mouse model of orthotopic HCC, leading to significant tumor regression [160]. Gao et al. [161] formulated sorafenib into CXCR4-targeted lipid-encapsulated PLGA NPs and modified them with a CXCR4 antagonist (AMD3100). The blocking of CXCR4/SDF1α via AMD3100 connected to the NPs led to a decrease in the infiltration of tumor-related macrophages, improvement in anti-angiogenesis, delay in tumor growth, and overall survival gain in orthotopic HCC mice.

### Thermotherapy with NPs

Thermotherapy accelerates DNA damage in cancer cells by increasing the temperature near these cells and destroying proteins essential for DNA repair [162]. However, its use is limited due to inadequate heating of tumor cells, reduced temperature dispersion and heat transmission, risk of organ damage from overheating, and difficulties in heat regulation that may cause bleeding [163]. Alternative methods have been developed to overcome these problems. For HCC cells grown in vitro, magnetic fluid hyperthermia using Fe₂O₃ NPs dramatically reduced their ability to proliferate, triggered apoptosis, and stopped the cell cycle in the G1/M phase [164]. Kandasamy et al. [165] reported the production of surface-functionalized and hydrophilic SPIONs and their use as nanomedicines for the treatment of HCC via magnetic fluid-based thermotherapy, which enhance killing HepG2 cells with efficiencies of 61–88% [165].

Notably, tumor cells tend to be more susceptible to radiation and chemotherapy when coupled with thermotherapy; moreover, temperatures above 46 °C accelerate the death of necrotic cells [166]. Therefore, NP-based thermotherapy may be a better and more effective therapeutic option for HCC.

### Photothermal therapy with NPs

Photothermal treatment (PTT) is a non-invasive technique that uses photothermal agents to transform light energy into heat that kills tumor cells [167]. When light triggers them at specific locations, these substances can eliminate cancer cells without harming healthy cells [168]. Recently, NPs have been utilized to introduce photosensitizers into tumor tissues, and copper sulfide NPs have been prioritized for PTT due to their low cost, simplicity of production, and high efficiency [169, 170]. The release of payload upon photoactivation relies on the co-loading of the intended agent with the photothermite in heat-sensitive NPs. Upon photoactivation, the encapsulated photothermite increases the temperature of the NPs, which triggers payload release [171].

Jin et al. [172] synthesized the multifunctional therapeutic agent SP94-modified polypyrrole-BSA-ICG NPs. This system combines photoacoustic/fluorescence imaging with cancer PTT to produce more tumor accumulation but lower nonspecific uptake in other healthy organs. Chang et al. [173] prepared DOX-loaded NPs modified by hematoporphyrin to increase light-guided PTT effectiveness for treating HCC. In an in vitro model, PTT nanotherapy with albumin-coupled gold NPs for solid HCC resulted in considerable necrosis of the malignant tissue, while the surrounding parenchymal tissue was not severely affected [174]. Ma et al. [175] encapsulated GPC3^+^ HCC cell-specific chimeric antigen receptor T cell membranes onto mesoporous silica that included IR780 NPs to prepare a novel nanomaterial with enhanced targeting and PTT abilities to induce apoptosis in 808-nm NIR-treated HCC cells. Nonetheless, most research has applied this strategy only in vitro or in animal models, necessitating extensive clinical studies to evaluate nanoparticle-packaged PTT efficacy.

### Radiotherapy with NPs

While radiotherapy is not typically utilized as the first-line therapy for HCC due to its low sensitivity, it is frequently employed as a palliative measure for advanced HCC to enhance the patients’ quality of life, particularly for those with bone metastases [176]. Radiation resistance induced by the hypoxic microenvironment in HCC is a major obstacle to clinical radiotherapy [177]. This restricts the sensitivity of radiation response detection along with the treatment rates for both healthy and tumor tissues, as healthy and cancer cells share many traits [178]. Through various pathways, NPs can increase the activity and lower the toxicity of radiation treatment by either targeting tumor cells to deliver radiosensitizers or enhancing image-guided radiation therapy [179]. NPs can also facilitate the combination of radiotherapy and chemotherapy to achieve synergistic effects while mitigating side effects. Additionally, they facilitate image-guided radiation therapy to improve precision and accuracy while reducing the exposure of the surrounding normal tissue [180]. To fully realize the potential of radiotherapy in cancer treatment, Zhang et al. [181] designed NBTXR3 to amplify the effects of radiotherapy and demonstrated that NBTXR3 could be universally used for treating various solid tumors when radiotherapy is indicated. AuNPs enhance the radiosensitization of HCC cells and increase DNA damage and apoptosis [182]. Stereotactic body radiation therapy is an emerging ablative therapy for HCC. A novel therapeutic agent based on Bi/Se NPs has been developed for CT image-guided stereotactic body radiation therapy sensitization in mice in vivo [183].

### Limitations and challenges in the application of NPs for HCC

Despite the promising use of NPs in treating HCC, the unique interactions between NPs and biological systems raise concerns regarding long-term biodistribution, potential toxicity, and unforeseen immune responses that complicate their clinical translation [184]. Researchers believe that prolonged exposure to NPs can damage the human body due to their superior penetrating power and unique “nano” size toxicity [185]. During immune stimulation, the interaction of NPs with specific cell surface receptors for leukocyte subpopulations and other innate immune cell components induces cytokine storms, interferon responses, and/or lymphocyte activation, resulting in adverse damaging effects collectively referred to as immunotoxicity [186]. Therefore, immunotoxicity studies are essential to evaluate any immune response induced by NPs [187], including the pseudo-allergic responses linked to particular NPs.

While numerous studies have explored issues such as cytotoxicity, NP dispersion, size-related toxicity, physiological responses, and retention times, their findings remain inconsistent [188]. Despite the mononuclear phagocyte system that leads NPs to the liver, which is the major location for treating HCC, conditioned NPs are directed to Kupffer cells in the liver. This process leads to the elimination of the NPs rather than exerting the intended pharmacological effects [189]. As such, drug delivery methods face significant challenges, including excessive carrier accumulation in the liver, reduced effectiveness against cancer cells, and barriers within vascular or tumor regions that hinder drug penetration into HCC cells.

The properties of NPs may also contribute to issues associated with increased intravascular friction and adhesions, potentially impacting atherosclerotic plaque formation and pathological changes [190]. Thus, the mechanism by which NPs cross the vessel wall and accumulate at the tumor site requires further investigation. Indirect evidence suggests that nanomedicines lose their original physicochemical properties upon entering the HCC tumor site and fail to inhibit tumor proliferation or migration in vitro. Moreover, the preferential accumulation of certain polymer-bound drugs in the kidneys may cause nephrotoxicity, with excipients potentially inducing additional unexpected toxicity [191]. Moreover, NPs can induce harm to the lungs and other organs and tissues, leading to apoptosis, DNA damage, inflammatory response, changes in the cell cycle, and aberrant gene expression [192]. Physical damage from nanocarriers is mainly due to their non-biodegradability, allowing them to accumulate and move freely within body tissues, leading to long-term physical damage [193, 194]. Long-term safety assessments are critical to the clinical translation of NPs. These assessments involve long-term exposure studies to monitor any chronic toxicity, including the potential for nanoparticles to induce tumor effects or chronic inflammation [195].

The manufacturing processes, reproducibility of large-scale production, and high production costs also pose challenges to the translation of nanocarrier-based therapies into clinical practice [196]. Additionally, financial and logistical obstacles that impede advocacy for supportive healthcare policies and equitable access to these cutting-edge therapies obstruct the widespread adoption of nanomedicines [197]. NPs have a dual role in both imaging and treatment; balancing the imaging agent with its therapeutic potential is a challenge, and the choice of imaging modality significantly impacts overall biocompatibility [198]. Drug encapsulation optimization, ligand splicing efficiency and high reproducibility of low-cost biomaterials are crucial for future clinical applications of nanotheranostic diagnostics.

### Clinical translational pathways

Barriers that limit the application of NP-based HCC therapy from the laboratory to the clinic include nanomaterial safety, scalability, regulatory approval, as well as patient stratification [199]. Developing NPs using biocompatible and biodegradable materials can minimize toxicity and increase safety. Targeting components can also be added to minimize off-target effects, which can now be achieved through state-of-the-art surface modification techniques and the application of biomimetic materials [200, 201]. Meanwhile, utilization of NP conjugates, such as vascular targeting molecules, may better enhance the ability of the NP system to selectively target and navigate tumor vasculature [202, 203]. Advanced manufacturing techniques using nanotechnology, along with continuous manufacturing processes and automation, can increase scalability and reduce costs [204]. Moving from laboratory research to market involves rigorous regulatory scrutiny, which can be lengthy and expensive. Engaging with regulators early in the development process could address regulatory challenges. Developing clear guidelines for nanomedicine evaluation could also simplify the process [205]. Addressing limitations and advancing integrated strategies by leveraging advances in nanotechnology, materials science, and interdisciplinary collaborations can help realize the full therapeutic potential of combining NP-based and traditional therapeutic interventions [206]. Patient selection should also be refined to identify individuals most likely to benefit from NP-based therapies, possibly using biomarkers and genetic analysis.

Clinical translation of nanomedicines requires careful consideration of NP building materials, size, surface properties, charge, drug loading/encapsulation efficiency, drug distribution, and metabolism and excretion of the carrier during application [207, 208]. Microfluidics enable high-speed self-assembly of nanomedicines with a narrower size distribution, tunable physical and chemical properties, and higher batch-to-batch reproducibility [209, 210]. Another promising technique is particle replication in nonwetting templates, which allows precise control of NP size, shape, chemical composition, drug loading, and surface properties to synthesize monodisperse nanoparticles [211, 212]. Recent advances in engineered NPs could facilitate the clinical translation of NP-based delivery systems and improve patient-specific therapeutic responses, focusing on the use of biomaterials and biomedical engineering innovations to overcome biological barriers and patient heterogeneity, enhancing drug uptake and accumulation at the tumor site, resulting in a significant increase in antitumor activity against H22 cells (HCC) [213–215]. Artificial intelligence can optimize multiple aspects of NP design, including NP size and charge, drug encapsulation efficiency, interactions with biofilms, blood vessels, biofluids, and drug release kinetics [216].

### Limitations of the review

Although our review of NPs and their interaction with various therapeutic options for treating HCC is timely, there are some limitations. Despite the encouraging preclinical results, translation to the clinical setting often results in differing effectiveness due to biological complexity and individual patient variability. There may be little or no consideration of the differences between different patient populations in existing studies. This may carry over into discussions of other regulatory mechanisms, signaling pathways, and NP properties that are crucial to understand but may be too general to provide meaningful guidance to researchers and clinicians. Moreover, while our article holds great promise for more targeted approaches based on patient and HCC characteristics, it is not currently possible to provide a clear roadmap for translating these innovations to clinical settings.

## Conclusion

The enhanced properties of NPs open new avenues for the diagnosis and treatment of HCC, a complex and heterogeneous disease for which conventional treatment proves to be challenging. NPs can be tailored to target HCC cells precisely, enhancing imaging agents that improve the sensitivity and accuracy of imaging techniques. This enables the detection of small tumors, facilitating early intervention and improved patient prognosis. NP surface engineering targets evading phagocytosis through RES, prolonging blood circulation and enhancing bioavailability. Furthermore, NPs can be designed to enhance tumor targeting and therapeutic responses by incorporating liver cancer’s microenvironment features. Certain NPs feature strong hepatic targeting, delayed release, and modifiability, which enhance therapeutic efficacy, reduce side effects, and increase liver drug concentrations. Furthermore, nanoengineering advancements offer opportunities to enhance patient-specific treatments and facilitate the practical implementation of NP-based delivery systems.

During HCC treatment, it is essential to consider potential damage to normal liver tissue. Safety tests should be conducted to assess the impact of various inorganic chemicals and foreign substances that may induce thrombosis, as well as the adverse effects and biotoxicity of chemotherapeutic drugs. The use of drug delivery NPs in medical therapeutics shows promise for enhancing and streamlining current clinical testing procedures. Despite the effectiveness of various NPs in delivering combination therapies to tumor cells, their clinical translation is challenging. Translating clinically feasible NP-based therapies can improve treatment outcomes while ensuring safety and efficacy, which is imperative for HCC treatment.

## Data Availability

No datasets were generated or analysed during the current study.

## Abbreviations

Au DENPs: Dendritic polymer-coated gold nanoparticles
CNMs: Carbon-based nanomaterials
CT: Computed tomography
Cur: Curcumin
DEN: Diethylnitrosamine
DOX: Doxorubicin
EPR: Enhanced permeability and retention
HCC: Hepatocellular carcinoma
HP: Hesperidin
ICG: Indocyanine green
miRNAs: microRNAs
MRI: Magnetic resonance imaging
NPs: Nanoparticles
NIR: Near-infrared
PAMAM: Polyamidoamine
PEG-MSN@ATO: PH-responsive nanoplatform
PEI: Polyethyleneimine
PLGA: Polylactic-co-glycolic acid
PMO: Periodic mesoporous organosilica
PTT: Photothermal treatment
SPIONs: Supermagnetic iron oxide nanoparticles
RES: Reticuloendothelial system
ROS: Reactive oxygen species
siRNAs: Small interfering RNAs
TME: Tumor microenvironment
US: Ultrasonography
TACE: Transarterial chemoembolization
VEGF: Vascular endothelial growth factor
